# Piloting a food photo sorting activity in Samoa to assess maternal beliefs and their role in child diet

**DOI:** 10.1111/mcn.12974

**Published:** 2020-02-14

**Authors:** Veeraya K. Tanawattanacharoen, Courtney C. Choy, Trevor J. Anesi, Take Naseri, Christina Soti‐Ulberg, Muagututia S. Reupena, Nicola L. Hawley

**Affiliations:** ^1^ Department of Chronic Disease Epidemiology Yale School of Public Health New Haven Connecticut; ^2^ Department of Epidemiology, International Health Institute, School of Public Health Brown University Providence Rhode Island; ^3^ Samoa Ministry of Health Apia Samoa; ^4^ Lutia i Puava ae Mapu i Fagalele Apia Samoa; ^5^ Yale Institute for Global Health Yale University New Haven Connecticut

**Keywords:** child diet, child nutrition, food sorting activity, maternal beliefs, nutrition knowledge, Samoa

## Abstract

Eating habits begin forming early in life when parental beliefs and behaviours often play a major role in shaping dietary intake. We aimed to assess maternal beliefs about the cost, social status, and nutritional value of foods in Samoa—a setting with an alarming burden of childhood obesity—and to determine how those beliefs may be related to child dietary intake. Samoan mothers (*n* = 44) sorted photographs of 26 foods commonly consumed in children in Samoa by cost, social status, and nutritional value (healthfulness). Responses were then assessed for their association with child dietary intake (reported using a food frequency questionnaire) using Pearson correlations. Mothers indicated that traditional Samoan foods were healthier, of higher social status, and lower cost compared with non‐traditional/imported food items. Compared with nutritional experts and a market survey of food prices, mothers demonstrated strong nutritional (*r* = .87, 95% CI [0.68, 0.95], *p* < .001) and consumer (*r* = .84, 95% CI [0.68, 0.93], *p* < .001) knowledge. The perceived cost of food was more strongly associated (*r* = −.37, 95% CI [−0.66, 0.02], *p* = .06) with child dietary intake than either healthfulness or social status, with decreasing consumption reported with increasing food cost. Our findings contradicted the notion that the high social status of imported foods may be contributing to increased intake and rising prevalence of childhood obesity in this developing country setting. Despite their nutritional knowledge, Samoan mothers may need additional support in applying their knowledge/beliefs to provide a healthy child diet, including support for access to reasonably priced healthy foods.

**Key Messages**

- Samoan mothers viewed traditional Samoan foods as healthier, of higher social status, and lower cost. The social status finding was contrary to hypotheses rationalizing the increasing prevalence of the modern diet.
- Samoan mothers have accurate nutritional and consumer knowledge when compared against nutritional experts and a survey of local food prices.
- Mother's perception of food cost, but not healthfulness or social status, was related to the frequency at which their child consumed the food item.
- The simple food photo sorting tool used here identifies key maternal beliefs about food, which may be important to consider in future nutritional intervention design.

## 1 INTRODUCTION

Samoa, a small island nation in the South Pacific, faces an alarming and rising burden of obesity, metabolic syndrome, and associated noncommunicable diseases (NCDs; Hawley & McGarvey, 2015; Ng et al., 2014; World Health Organization, 2002). A survey of 25‐ to 64‐year‐old Samoan adults over the past three decades revealed that obesity (body mass index [BMI] ≥30 kg/m^2^) rose from 27.7% to 53.1% among men and 44.4% to 76.7% among women, while the prevalence of Type II diabetes rose from 1.2% to 19.6% among men and 2.2% to 19.5% among women (Lin et al., 2017). Although the increasing prevalence has been well‐documented among adults, the burden of overweight/obesity has been less studied among Samoan children. Recent data from the *Ola Tuputupua'e* (Growing Up) study, following the growth and development of a cohort of ~500 Samoan children, estimated that 16–30% of 2‐ to 4‐year‐olds were overweight/obese (Choy et al., 2017), indicating that interventions to reduce obesity risk must begin early in life.

Researchers have attributed the increase in prevalence of obesity and related NCDs among the Samoan population to a nutritional transition, characterized by urbanization, a shift towards sedentary lifestyle and employment, and modernization of traditional diets (Keighley et al., 2007; Popkin, 1998). In the past half century, food imports to Samoa have increased fivefold resulting in an influx of processed foods (high in energy density, refined sugar, white flour, trans fat, polyunsaturated fats, and salt) and a 47% increase in available energy from food (DiBello et al., 2009; FAOSTAT, 2002; Seiden, Hawley, Schulz, Raifman, & McGarvey, 2012; Shridhar et al., 2015). Following a ‘modern’ diet has been associated with obesity and metabolic syndrome in Samoan adults and obesity in children (Choy, 2018; DiBello et al., 2009; Wang et al., 2017).

A systematic literature review of 25 studies around the world concluded that children who are overweight/obese have an increased risk of remaining overweight/obese as adults (Singh, Mulder, Twisk, Van Mechelen, & Chinapaw, 2008). This trend may reflect the tendency for dietary habits formed in early childhood to persist into late childhood and adolescence (Kelder, Perry, Klepp, & Lytle, 1994; Skinner, Carruth, Bounds, & Ziegler, 2002). Early childhood is also a unique transitional period during which parents hold great influence over their child's food behaviours and diet (e.g., food selection, preferences, and portion sizes; Scaglioni, Salvioni, & Galimberti, 2008; Watkins & Jones, 2015). Understanding the factors that influence parental feeding behaviour is essential to shaping a healthy early child diet. A study in neighbouring American Samoa explored mothers' attitudes and beliefs about infant feeding, with a particular focus on breastfeeding (Hawley et al., 2015), but mothers' attitudes and beliefs as they pertain to early childhood diet have yet to be explored.

Studies conducted in other settings have focused on maternal nutritional knowledge, finding some associations with child diet, but few have examined the role of additional factors such as the cost and social value of foods. Among Omani and Flemish mothers, lower maternal nutritional knowledge was associated with lower dietary adequacy for preschool‐aged children (Al‐Shookri, Al‐Shukaily, Hassan, Al‐Sheraji, & Al‐Tobi, 2011; Vereecken & Maes, 2010). In Mozambique, high maternal nutritional knowledge led to a more diversified diet for their preschool‐aged child (Burchi, 2010). In Ghana and Lesotho, maternal nutritional knowledge of infant feeding was associated with infant nutritional status (Appoh & Krekling, 2005; Ruel, Habicht, Pinstrup‐Andersen, & Gröhn, 1992). However, in socio‐economically disadvantaged neighbourhoods in Victoria, Australia, researchers were unable to find an association between maternal nutritional knowledge and preschool‐aged child diet; they hypothesized that limited resources and cost of foods may have played a larger role (Williams, Campbell, Abbott, Crawford, & Ball, 2012). Understanding child diet is additionally complex in Samoa because meals can be shared with nuclear and extended family members sometimes in multiple households. In this context, additional structural and cultural factors may influence what Samoan mothers feed their children.

To address current gaps in knowledge and inform future intervention strategies, the following study examines maternal beliefs surrounding food healthfulness, social status, and cost as well as maternal nutritional and consumer knowledge as they relate to early child dietary intake.

## 2 METHODS

### 2.1 Setting

Samoa is an upper‐middle‐income country with an estimated annual gross domestic product per capita of US$4,393, which is approximately 11,550 Western Samoan Tala (WST; Samoa Bureau of Statistics, 2018a). It is composed of two main islands, Upolu and Savai'i, and several islets. According to the 2016 census, the majority (77.8%) of the 195,979 people inhabiting Samoa live in Upolu (Samoa Bureau of Statistics, 2016a). The two official languages are Samoan and English, and the literacy rate is 97%.

### 2.2 Sampling

This pilot study was embedded within the ongoing *Ola* Tuputupua'e study cohort designed to observe the growth, development, and onset of cardiometabolic diseases among Samoan children. The cohort includes a convenience sample of 319 Samoan mother–child pairs, first recruited June to August 2015, and an additional 179 pairs, added to the sample between June and August 2017 (total *n* = 498; Choy et al., 2017). Because one of the original aims of the study was to examine whether urbanization and exposure to the nutrition transition was associated with growth and development, the original sample was not nationally representative, but contained an approximately equal proportion of participants from all three census regions in Upolu (Choy et al., 2017; Samoa Bureau of Statistics, 2016a). There are five study villages in the Apia Urban Area (urban region; high exposure to nutrition transition), three in Northwestern Upolu (peri‐urban region; medium exposure), and three in the Rest of Upolu (rural region; low exposure). Eligible participants include healthy children of maternal‐reported Samoan ancestry (four Samoan grandparents) who were 2 to 4 years old in 2015 or 4 to 7 years old in 2017 and mothers without severe physical or cognitive impairments, who were not pregnant at the time of recruitment.

The subsample of participants included in this analysis was surveyed between June and August 2018. During the 2017 data collection wave, we were unable to contact 81 of the study families because they were unavailable at the time of the survey, had moved out of their original study village, or were lost to follow up. An attempt was made in 2018 to locate these families, and *n* = 49 of the families (with children now aged 5–8 years old) participated in a follow‐up assessment. Of the 49 follow‐up appointments, 44 mothers completed the food photo sorting activity.

Mayors and women's committee representatives of study villages served as liaisons between the research team and the study participants to assist with locating participants. In the follow‐up assessments conducted at the participants' homes, a local Samoan enumerator explained the study procedures, potential risks and inconveniences, benefits, economic considerations, treatment alternatives, privacy/confidentiality, and voluntary participation and withdrawal in the participant's preferred language (typically Samoan). Written informed consent was obtained from the mothers. For the follow‐up appointments, if children were 7 years of age or older, Samoan enumerators explained the study to them using language they could understand and additional written assent was obtained from the child per Institutional Review Board Protocol (Choy et al., 2017).

### 2.3 Ethical consideration

The *Ola Tuputupua'e* Study was approved by the Yale Institutional Review Board (Human Investigation Committee #2000020519), the Samoa Ministry of Health Health Research Committee, and Brown University (Institutional Review Board Authorization Agreement #18‐41, Protocol 959).

### 2.4 Child, maternal, and household characteristics

Child age, sex, and schooling status, maternal age, level of education, and employment and household census region, size and annual income, and ownership of a home plantation for personal use were assessed by the same questionnaire as prior waves of the *Ola Tuputupua'e* Study (Choy et al., 2017). Mothers reported all child characteristics, their highest level of education, and their employment status. Census region reflected urbanization and was determined from the child's village of residence, which was not always the same as their mothers. Mothers reported household size to include all people who lived in the household over the past 6 months. In reporting annual household income, which may impact food‐related spending power, mothers were prompted to include income from work, family overseas, retirement, and other sources added together. If mothers indicated that either themselves or another household member living in their home “spent time doing activities in the garden/lawn/plantation for personal use,” they were reported as having a home plantation for personal use in this study, which was included in analyses because several of the foods of interest are commonly grown in Samoa.

In order to describe the characteristics of the sample, child weight and height measurements were taken wearing light island clothing and no footwear. The World Health Organization child growth standards and references were used to identify overweight and obesity for children based on a BMI‐for‐age *z*‐score >+2 SD for under 5 years and >+1 SD for ages 5–7 years (World Health Organization, 2007).

### 2.5 Child dietary intake

Child dietary intake was measured by a 115‐item food frequency questionnaire (FFQ) adapted for Samoan children. This tool was validated among Samoan adults and adapted for use among children in 2015 and 2017 (Choy, Wang, et al., 2018; DiBello et al., 2009). Mothers completed the FFQ for their child with the exception of one whose child gave responses while she supervised. This child's reported intake was checked and included in the analysis as it reflected the sample means. The FFQ recorded the frequency of intake for each food item; the options included never or <1/month, 1–3/month, 1/week, 2–4/week, 5–6/week, 1/day, 2–3/day, 4–5/day, and ≥6/day. Following previously described methodology, calories from each food item were calculated by converting all frequencies to daily frequency, multiplying by a fixed portion size, and multiplying by caloric density (Choy, Thompson, et al., 2018). In analyses, the frequency of intake and percentage of daily calories from each food item (calories from food divided by total caloric intake) were used to assess child intake of each food item.

### 2.6 Maternal food beliefs

While maternal nutritional knowledge has been historically assessed using a questionnaire about nutritional behaviours such as breastfeeding, this study used the relative rankings of staple Samoan foods to quantify maternal beliefs. It repurposes a qualitative food photo sorting activity by taking a quantitative analysis approach (Hesketh, Waters, Green, Salmon, & Williams, 2005).

Using FFQ data from *Ola Tuputupua'e* children visited in 2017, the most frequently consumed foods across five categories (fruits and vegetables, carbohydrates, protein, “processed” foods, and drinks) were identified for use as the photographic prompts in this study. A total of 27 food photo cards—26 individual food times and 1 aggregate of fruit and vegetables—were made on laminated cardstock. The fruits and vegetables selected were eggplant, laupele (local spinach), peppers, pumpkin, apples, bananas, oranges, and papayas. The carbohydrates selected were breadfruit, taro, bread, and rice. The proteins selected were eggs, grilled chicken, fried chicken, boiled fish, fried fish, tinned fish, and tinned fish in tomato sauce (this item appears in figures as ‘tinned fish TM’). The processed foods selected were fried pancake balls (*panikeke*), fries, ice cream, and pizza. Finally, the drinks selected were coffee/tea, soda, and water. All produce (e.g., fruits and vegetables) and eggs were depicted in their raw forms, and all other items were shown in their commonly eaten forms.

Mothers were asked to rank 19 cards by healthfulness (least to most healthy) and 26 cards by social status (lowest to highest status) and perceived cost (lowest to highest cost). In the healthfulness rank, the individual fruit and vegetable cards (*n* = 8) were replaced by an aggregate photo of many fruits and vegetables to reduce time because prior qualitative work has shown that Samoan mothers consistently identify fruits and vegetables as being healthy (Choy, 2016). In the social status sort, mothers were additionally prompted “foods with higher social status are ones you would most likely bring to visit family at home or in the hospital or to bring to Sunday *to'onai*,” (a traditional family lunch gathering, which occurs every Sunday after church).

### 2.7 Expert and standard ranks

An expert ranking of food healthfulness was developed by surveying nutritionists and registered dietitians (*N* = 8, from Samoa [n = 2] and the United States [n = 6]). Experts were asked to rank the same 19 foods as the participants from least to most healthy. Their rankings were averaged to create an expert rank. A standard rank of food cost was developed by surveying food prices in the main outdoor market and the two largest grocery stores in Apia (capital city of Samoa) in August 2018. The various prices of a single food item at all three locations were averaged, and the final standard cost rank was in terms of WST per volume (grams). Foods, which are typically home grown were equally ranked as the lowest cost. Healthfulness and cost misconception scores were calculated by taking the difference between each mother's rank and the expert or standard rank for all food items in the perceived healthfulness and cost sorting schemes. The greater the difference, the more disparate a mother's ranking was from the constructed standard, which was analysed to reflect increasing degrees of misconceptions surrounding food health and cost.

### 2.8 Statistical methods

Individual mother's rankings for each food item in each sorting scheme were averaged and then re‐ranked to create aggregate ranks. A rank of 1 represented the lowest perceived healthfulness, social status, and cost, whereas a rank of 19 for the healthfulness sort, and a rank of 27 in the social status and cost sorts represented the highest. Correspondence between aggregate maternal rankings, maternal versus standard rankings, and maternal rankings versus child intake were examined using Pearson correlations. Socio‐demographic correlates of maternal rankings and child intake were also identified using Kruskal–Wallis H test. R Studio version 3.5.1 (R Foundation for Statistical Computing, Vienna, Austria) and SAS 9.4 Software (SAS Institute Inc., Cary, North Carolina) were used for all analyses.

## 3 RESULTS

### 3.1 Characteristics of the study population

The subset sampled was generally representative of the socio‐demographic distribution of the *Ola Tuputupua'e* cohort (Table 1; Choy et al., 2017). The mean age of the children was 5.94 ± 0.97 years. The cohort of children was evenly divided by sex (43.2% female and 56.8% male), and most of the children (84.1%) had started school. The mean age of the mothers was 34.3 ± 7.8 years, and the majority (79.5%) were unemployed. The majority of households surveyed (68.2%) reported having a plantation at their residence for personal use.

**Table 1:** Characteristics of the study population

Characteristic	Mean (*SD*) or percentage
Child (*n* = 44)	
Mean age (years)	5.9 (1.0)
Female	43.2
Male	56.8
Formal schooling	
No	15.9
Yes	84.1
Overweight	29.6
Obese	6.8
Mother (*n* = 48)	
Mean age (years)	34.3 (7.8)
Highest level of education	
Primary school	4.5
High school	72.7
University	22.7
Employment	
Unemployed	79.5
Full time	18.2
Maternity leave	2.3
Household	
Census region	
Apia Urban Area	22.7
Northwestern Upolu	40.9
Rest of Upolu	36.4
Mean household size	8.2 (3.4)
Household income (WST)	
Less than 10,000	6.8
10,000–30,000	63.6
More than 30,000	29.6
Home plantation for personal use	68.2

Abbreviations: *SD*, standard deviation; WST, Western Samoan Tala.

### 3.2 Maternal beliefs surrounding the healthfulness, social status, and cost of traditional foods

Aggregating all mothers' rankings revealed that, as a group, the mothers believed fruits and vegetables were the healthiest food item, while viewing soda as the least healthy (Table 2). Across the entire sample of mothers, boiled fish was ranked as the healthiest source of protein whereas fried chicken was the least healthy and taro, closely followed by breadfruit, as the healthiest source of complex carbohydrates whereas fries was the least healthy. The foods considered to have the highest social status were oranges, followed by papaya and apple, whereas the lowest were ice cream, fried pancake balls, and soda. The foods perceived to be the most expensive were pizza, fried chicken, and fries, whereas the least expensive were banana, papaya, laupele, and water. On the island, taro, breadfruit, carrots, eggplant, pumpkin, laupele, bananas, and papaya are grown commonly on home plantations and often consumed at no monetary cost for families who have home plantations (Samoa Bureau of Statistics, 2018b). This was reflected in the aggregate cost rankings. Traditional foods, as defined by DiBello et al. (2009) are marked with letters in Table 2.

**Table 2:** Mother's (*n* = 44) average rankings of frequently consumed foods^a^

	Healthfulness	Social status	Perceived cost
Highest	Fruits and vegetables^a^	Orange	Pizza
	Boiled fish^a^	Papaya^a^	Fried chicken
	Taro^a^	Apple	Fries
	Breadfruit^a^	Banana^a^	Grilled chicken
	Water	Taro^a^	Fried fish
	White rice	Boiled fish^a^	Boiled fish^a^
	Fried fish	Pumpkin^a^	Tinned fish
	Eggs	Carrots^a^	Ice cream
	Tinned fish	Peppers	White rice
	White bread	Laupele^a^	Carrots^a^
	Tinned fish in tomato sauce	Eggplant^a^	Eggs
	Grilled chicken	Breadfruit^a^	Soda
	Tea and coffee	Fried fish	Tinned fish in tomato sauce
	Pizza	Fried chicken	Peppers
	Fried pancake balls	Grilled Chicken	Pumpkin^a^
	Fried chicken	Pizza	Orange
	Fries	Water	Apple
	Ice cream	Eggs	Taro^a^
	Soda	White Rice	Eggplant^a^
	—	Fries	Fried pancake balls
	—	Tinned fish	White bread
	—	Tinned fish in tomato sauce	Breadfruit^a^
	—	White bread	Tea and coffee
	—	Tea and coffee	Banana^a^
	—	Ice cream	Papaya^a^
	—	Fried pancake balls	Laupele^a^
Lowest	—	Soda	Water

a Traditional foods.

**Figure 1:**
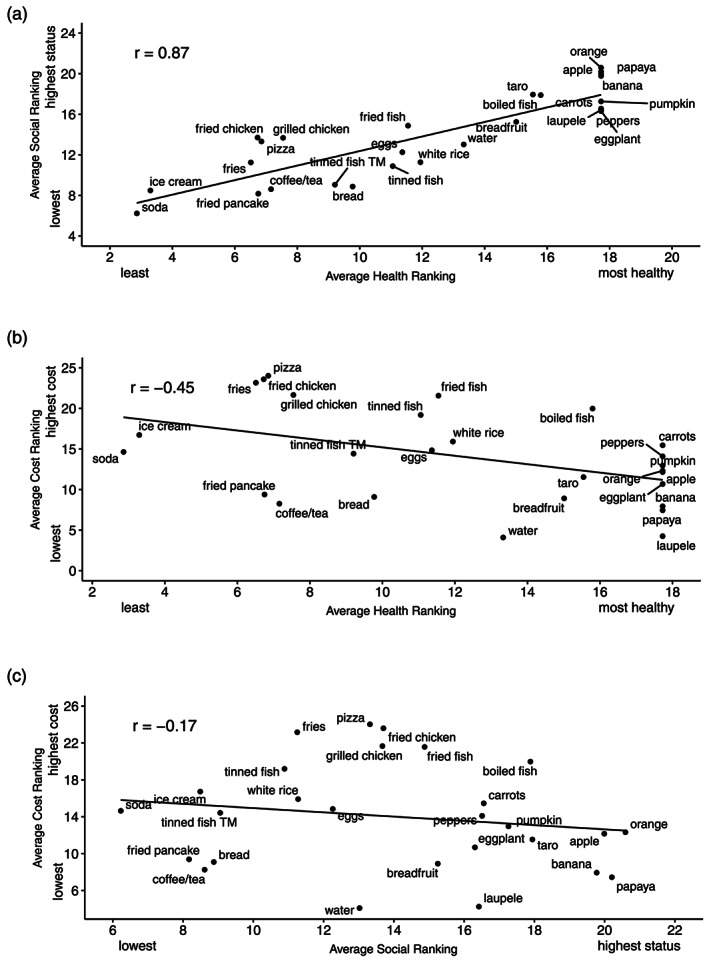
The average of mother's (*n* = 44) rankings of each food item in each sorting scheme are plotted. The trend line represents the correlation between average mother's (a) health and social status ranking (*r* = .87, 95% CI [0.72, 0.94], *p* < .001), (b) health and cost rankings (*r* = −.45, 95% CI [−0.71, −0.09], *p* = .02), and (c) social status and cost rankings (*r* = −.17, 95% CI [−0.51, 0.23], *p* = .41)

Bivariate analysis of the aggregate rankings revealed that mothers' ranking of food healthfulness was strongly, positively correlated with social status (*r* = .87, 95% CI [0.72, 0.94], *p* < .001) and moderately, negatively correlated with perceived cost (*r* = −.45, 95% CI [−0.71, −0.09], *p* = .02; Figure 1a,b). Social status and perceived costs were not statistically significantly correlated (*r* = −.17, 95% CI [−0.51, 0.23], *p* = .41; Figure 1c). Locating traditional foods in these correlations reveal that traditional foods are viewed as healthier, of higher social status, and of lower perceived cost than non‐traditional/imported ones.

### 3.3 Maternal nutritional and consumer price knowledge

**Figure 2:**
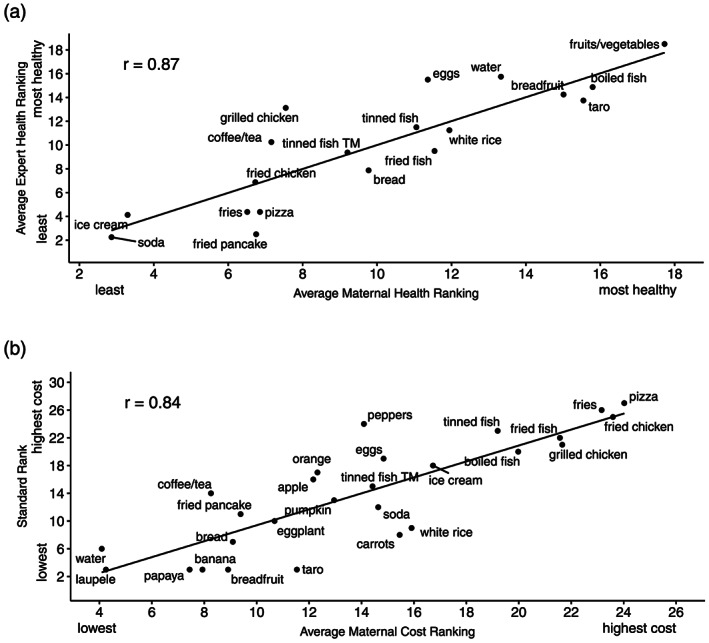
(a) Correlation between average mother's (*n* = 44) and average expert's (*n* = 8) health rankings (*r* = .87, 95% CI [0.68, 0.95], *p* < .001). (b) Correlation between average mother's (*n* = 44) food cost rankings and a cost by volume (grams) ranking determined by market survey (*r* = .84, 95% CI [0.68, 0.93], *p* < .001)

Mothers' average health rankings (*r* =0.87, 95% CI [0.68, 0.95], *p* < .001) and cost rankings (*r* = .84, 95% CI [0.68, 0.93], *p* < .001) were strongly, positively correlated with the rankings made by nutritional experts and the market survey, respectively (Figure 2). However, mothers tended to rank bread, fried pancake, pizza, and taro as healthier and eggs, grilled chicken, and water as less healthy than experts did (Figure S1a). They also tended to overestimate the cost of bananas, carrots, papayas, rice, and taro, while underestimating the cost of apples, coffee/tea, eggs, fries, oranges, pizza, and tinned fish (Figure S1b).

### 3.4 Correlation between mother's food beliefs and their child's diet

**Figure 3:**
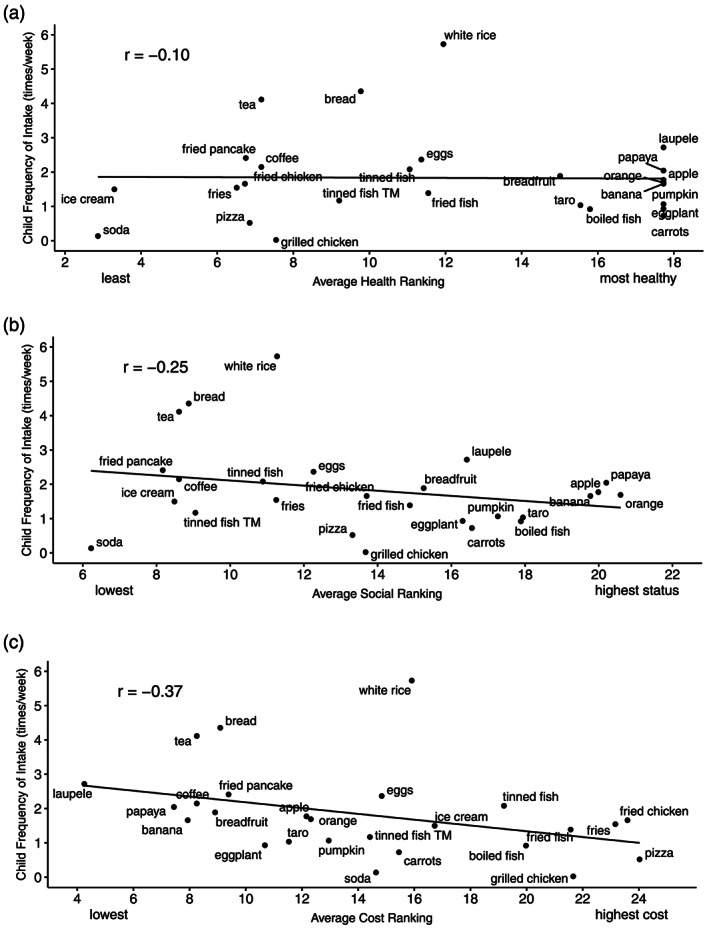
(a) Correlation between average mother's (*n* = 44) health rankings (*r* = −.10, 95% CI [−0.40, 0.38], *p* = .95), (b) social status rankings (*r* = −.25, 95% CI [−0.58, 0.15], *p* = .21), and (c) cost rankings (*r* = −.37, 95% CI [−0.66, 0.02], *p* = .06) and average child's (*n* = 44) weekly frequency of food intake

Mothers' perceived food cost (*r* = −.37, 95% CI [−0.66, 0.02], *p* = .06) was related to their child's usual frequency of food intake (Figure 3a), but food healthfulness (*r* = 0.10, 95% CI [−0.40, 0.38], *p* = .95) or social status (*r* = −0.25, 95% CI [−0.58, 0.15], *p* = .21) were not (Figure 3b,c). The more expensive mothers ranked a food item, the less frequently they reported that their child ate it. Foods that did not follow this pattern were bread, tea, and white rice. Bivariate correlations of individual mother's rankings of each food item and percentage of their child's daily caloric intake from each food item revealed limited significant associations (Table S1).

### 3.5 Socio‐demographic correlates of mother's rankings and their child's diet

Participant socio‐demographic characteristics were further explored for their correlation with mother's food ranking and their child's diet. Census region was associated with health rankings of taro, water, and fried fish and the percent of daily calories from rice, fries, and pizza. Mothers in more urbanized settings were more likely to rank taro, water, and fried fish as less healthy, and children in more urbanized settings were more likely to consume fries and pizza and less likely to consume fried fish. Annual household income was collapsed into a dichotomous variable, less than 30,000 WST or greater than or equal to 30,000 WST. Mothers of lower income (<30,000 WST) ranked the social status of eggplant, pumpkin, and papaya as lower than mothers of higher income. Mothers of lower income ranked the cost of taro as more expensive and the cost of soda and friend pancake balls as less expensive than mothers of higher income. Children of lower income households had a higher percentage of daily calories from taro and eggplant and a lower percentage from eggs, fries, and pizza as compared with children of higher income. Mothers with home plantations ranked the health of grilled chicken higher and the cost of taro lower than mothers without a home plantation. Although the highest level of mothers' education was not associated with their rankings for cost and social status of foods, mothers that completed a university education ranked the health of water higher compared with those who completed high school and/or primary school. Few associations were observed between mother's education and child's diet. The only significant findings mirrored those reported for income where children who had a mother that completed a university education had a higher percentage of daily calories from fries and pizza compared with those who did not.

## 4 DISCUSSION

Despite the fact that child food preferences are often established early in life and that parental beliefs and attitudes towards food likely play a major role in establishing those preferences, there had been no previous attempt to characterize parental beliefs about food in Samoa, a setting with prevalent childhood obesity. This study addresses that gap by documenting beliefs about commonly consumed foods and nutritional knowledge among Samoan mothers. Repurposing a previously qualitative food sorting task, we were able to demonstrate how maternal beliefs were related to child dietary intake, noting that (a) traditional foods were thought to be healthier, of higher social status, and lower cost than non‐traditional/imported foods, (b) that mothers had accurate nutritional and consumer knowledge, and (c) that cost of food influenced child intake, whereas healthfulness and social status did not.

The increasing consumption of a modern diet, enabled by the fivefold increase in imported foods over the past half century, suggested that imported foods had attained a higher social status than traditional foods among Samoans (DiBello et al., 2009; FAOSTAT, 2002). The mothers' contradictory ranking of traditional foods as healthier and of higher social status may be a product of recent eating health and economic promotion campaigns in both Samoa and the wider Pacific region where there has been a renewed focus on consumption of local produce for health. The Samoan Ministry of Health's 2016 “Eat the Rainbow” initiative encourages the consumption of a variety of local fruits and vegetables, and the Samoan Women in Business Development endorses organic produce to stimulate the local economy (“Samoa Observer, 2016”; “Women in Business Samoa, n.d.”; World Bank, 2018). In addition, the Secretariat for the Pacific Community recently released the first *Pacific Guidelines for a Healthy Diet and Lifestyle*, which placed emphasis on consuming local produce and traditionally grown foods for good health (Public Health Division of the Pacific Community, 2017).

This study finds that, on average, Samoan mothers have largely accurate knowledge of the relative healthfulness and cost of staple food items for the most part independent of mothers' highest level of educational attainment. Therefore, lack of knowledge seems to not be a barrier to healthy child diet. However, examining individual mother's rankings revealed some potential gaps in nutritional and consumer knowledge, which could still inform future public health action.

Mothers tended to overestimate the healthfulness of carbohydrate‐rich foods, in particular, bread and pizza compared with expert rankings. Mothers also tended to overestimate the healthfulness of fried foods (fried pancake balls) while underestimating the healthfulness of unfried foods (grilled chicken). However, alternative explanations may exist. For instance, grilling is an uncommon food preparation method and therefore, mothers may be unfamiliar with it. In general, the stock photos used for the sorting activity may have looked unfamiliar; however, the photos were selected with the help of our local enumerators. Another alternative explanation could also be portion size. While the healthfulness of fried pancake balls was overestimated, the healthfulness of fried chicken was more accurate. A local nutritional expert explained that Samoans view fried pancake balls as a light snack and therefore may be misestimating the amount of oil they consume when eating it. Public health education on the healthfulness of processed versus unprocessed carbohydrates and methods of food preparation may be beneficial.

In terms of cost misconception, mothers tended to overestimate the price of local produce while underestimating the price of imported produce. This suggests that efforts could be made to inform mothers of the cost effectiveness of growing and/or buying locally grown foods where time and other resources allow. A pilot study completed among mothers and children living in socio‐economically disadvantaged neighbourhoods in Victoria, Australia, demonstrated that a brief 10‐min slideshow was an effective educational intervention targeting perceptions of healthy food affordability (Williams et al., 2014). This intervention could be adapted for use in Samoa. However, one limitation to this specific analysis that should be noted is that we observed mothers collectively placing fruit and vegetable cards at the lowest end of the cost spectrum. They sometimes sorted between them arbitrarily or ranked them as equal in cost. Yet another alternative explanation is the price instability of local produce due to seasonal variations and periodic natural disasters (Samoan Ministry of Agriculture and Fisheries, 2011, 2016; Seiden et al., 2012).

In this sample, cost, but not healthfulness or social status, was modestly correlated with child diet. In this setting where the average weekly expenditure on food is $65.35 WST per capita per week compared with the average income that ranges from $25.60 to $103.70 WST per capita per week, costs of food, and not healthfulness or social status, may be the most salient factor in mothers' feeding decisions. This inverse relationship between food cost and frequency of child intake aligns with the existing literature, particularly of low socio‐economic status populations. Previous qualitative studies have identified perceived food cost as a primary factor influencing healthy eating and food choice among low‐income households (Darmon & Drewnowski, 2015; Inglis, Ball, & Crawford, 2005), and an inverse relationship between food prices and intake has been found in studies across North America, Europe, and Australia (Lee, Ralston, & Truby, 2011). In fact, other studies suggest that food cost mediates the association between low socio‐economic status and poor diet quality (Aggarwal, Monsivais, Cook, & Drewnowski, 2011; Inglis et al., 2005). Future studies might examine if this phenomenon is occurring in Samoa.

The true strength of the correlation between perceived food cost and child diet may be underestimated by the presence of outlying foods—white rice, bread, and tea—which seem to be most highly consumed among children regardless of their perceived cost. The cost misconception plots reveal that the cost of white rice—the greatest “outlier”—is overestimated by approximately 86% of Samoan mothers sampled (Figure S1b). This may be because white rice is sold in bulk, causing mothers to recall the price of an entire bag rather than a single serving. If white rice were removed from analysis, the correlation between average maternal cost ranking and child intake would become much stronger (*r* = −.53, 95% CI [−0.76, −0.16], *p* = .007).

The negative correlation between food cost and child intake seems to be discordant with the finding that traditional foods were ranked as healthier and less expensive, suggesting that cost was not a barrier to accessing healthy foods. Many of the least expensive foods were those which are commonly grown at little to no cost in home plantations. One explanation for this discrepancy may be that cost is only a barrier to accessing healthy foods, which are purchased rather than grown.

The paucity of correlations between individual mother's rankings of food healthfulness, social status, and cost and their child's intake of the food item might mean that other unmeasured variables were at play. In other studies, household availability of fruits and vegetables, salty foods, and soft drinks have been found to influence children's intake of these foods (Campbell et al., 2013; Cullen et al., 2001). Parental rules, modelling, and restrictions have also been shown to influence child dietary intake; rules around eating a certain amount of fruits and vegetables and maternal consumption of fruits and vegetables have a positive effect on child consumption while a systematic literature review found that parental feeding restrictions are associated with increased eating and childhood weight (Faith, Scanlon, Birch, Francis, & Sherry, 2004; Jones, Steer, Rogers, & Emmett, 2010).

The novel quantitative analysis of this food photo sorting activity has great potential for use in other populations. It could assess food beliefs in other contexts by simply replacing the staple Samoan food items with those that are most common in the population of interest. Additional culturally relevant maternal belief systems, beyond the ones measured here, could also be assessed by changing the sorting prompts. This tool, first developed by Hesketh et al. (2005) for use among children, is completely pictorial with no literacy requirements therefore making it accessible to almost all participants. The straightforward analysis of food rankings has the potential to further the understanding of maternal food beliefs and nutritional knowledge in populations for which little is known and who may bear the highest burdens of overweight/obesity and NCDs.

### 4.1 Limitations

The primary limitation of this pilot study is the small sample size, which limits the analyses that can be undertaken and the generalizability of these findings. With 44 observations, analyses were underpowered to detect correlations between maternal food beliefs and child weight status. Our sample was, however, characteristic of the general Samoan population in terms of maternal education, employment status, household size, and income (Samoa Bureau of Statistics, 2014, 2016b). Social desirability bias may have influenced the mother's responses, particularly in the strong, positive correlation between food healthfulness and social status. Mothers may have felt pressure to indicate that they valued healthier foods because it was clear that the study team was focused on healthy child growth and development. Moreover, because the concept of “social status” was grounded in many different examples (e.g., family visit, hospital visit, and Sunday *to'onai*), some mothers may have focused on one scenario rather than thinking broadly. Specifically, it might have been that mothers who focused heavily on what they would bring to the hospital placed fruits at a higher social status. Although the health and cost misconception scores provide useful comparison between mothers' rankings and expert and standard rankings, they also represent conversion of a relative measure into an absolute measure and therefore must be interpreted tentatively. While the FFQ has provided useful information about this cohort, we have observed a tendency for over‐reporting of dietary intake.

Future research should aim to survey a larger sample size to increase statistical power. This may reveal specific food items where child intake is strongly associated to maternal beliefs and allow associations between maternal beliefs and child weight status to be identified. Moreover, surveying mothers and children repeatedly over time will determine whether these maternal beliefs influence child dietary behaviour and, in turn, influence nutritional outcomes such as overweight/obesity.

## 5 CONCLUSION

Using a simple food photo sorting activity, we were able to document maternal beliefs about the nutritional value, social status, and cost of foods commonly consumed by Samoan children. Although we provide evidence that locally grown, traditional Samoan foods are favoured by mothers and believed to be healthier, only the cost of food was associated with child dietary intake, indicating the continued promotion as a strategy for improving child nutritional intake must be supported with actions to increase access to reasonably priced healthy food options.

## CONFLICTS OF INTEREST

The authors declare that they have no conflicts of interest.

## CONTRIBUTIONS

VKT was responsible for study design under the supervision of NLH. VKT, CCC, and TJA conducted the research with the support of TN, CS and MSR. VKT, and NLH analysed and interpreted the data. VKT drafted the manuscript. All authors reviewed and edited the final manuscript.

## Supporting information


**Figure S1.** (A) Distribution of mother's (n = 44) health misconception scores. Differences were calculated by subtracting the expert's ranking (1‐19) from the mother's ranking (1‐19). (B) Distribution of mother's (n = 44) cost misconception scores. Differences were calculated by subtracting the standard rank (1‐26) from the mother's ranking (1‐26). Positive differences can be interpreted as mothers ranking an item as healthier or more expensive than the standard.

Table S1. Spearman correlations between maternal rankings and % of child's daily caloric intake.^a^
