# Supplementary material for: Piloting a food photo sorting activity in Samoa to assess maternal beliefs and their role in child diet
Source: Matern Child Nutr. 2020 Feb 14;16(3):e12974. doi: 10.1111/mcn.12974 (PMC7296816; doi:10.1111/mcn.12974)
Supplement: Supplementary file 2 — Table S1. Spearman correlations between maternal rankings and % of child's daily caloric intake.a [file MCN-16-e12974-s001.docx]

**Supplementary Figures and Tables**

Supplementary Figure 4 is attached separately as individual files and the legend is included in the main file.

| Table 3. Spearman correlations between maternal rankings and % of child’s daily caloric intake.^a^ | | | | | | | | |
| --- | --- | --- | --- | --- | --- | --- | --- | --- |
| **Health**  **Rank** |  | **Healthfulness** | | **Social Status** | | **Perceived Cost** | | **Mean times eaten/month** |
|  |  | **r_s_** | **p** | **r_s_** | **p** | **r_s_** | **p** |  |
| **Highest** | **Carrots** | -- | -- | 0.247 | 0.11 | 0.123 | 0.428 | 3.16 |
|  | **Eggplant** | -- | -- | 0.134 | 0.387 | -0.18 | 0.246 | 4.05 |
|  | **Laupele (spinach)** | -- | -- | 0.21 | 0.172 | -0.15 | 0.331 | 11.84 |
|  | **Pumpkin** | -- | -- | 0.109 | 0.482 | -0.13 | 0.425 | 4.65 |
|  | **Apple** | -- | -- | 0.122 | 0.431 | -0.17 | 0.284 | 7.72 |
|  | **Banana** | -- | -- | -0.1 | 0.524 | -0.08 | 0.624 | 7.23 |
|  | **Orange** | -- | -- | -0.31 | 0.041** | -0.1 | 0.526 | 7.37 |
|  | **Papaya** | -- | -- | -0.06 | 0.698 | 0.037 | 0.816 | 8.91 |
|  | **Boiled fish** | 0.048 | 0.758 | -0.12 | 0.442 | -0.04 | 0.792 | 4.01 |
|  | **Taro** | -0.03 | 0.817 | -0.13 | 0.407 | -0.05 | 0.776 | 4.51 |
|  | **Breadfruit** | -0.02 | 0.91 | 0.252 | 0.099* | -0.15 | 0.326 | 8.22 |
|  | **White rice** | -0.09 | 0.576 | 0.08 | 0.607 | 0.222 | 0.147 | 24.96 |
|  | **Fried fish** | -0.17 | 0.26 | -0.06 | 0.715 | 0.209 | 0.174 | 6.05 |
|  | **Eggs** | 0.298 | 0.049** | 0.209 | 0.173 | -0.08 | 0.619 | 10.31 |
|  | **Tinned fish** | 0.233 | 0.128 | 0.219 | 0.153 | 0.064 | 0.682 | 9.06 |
|  | **White bread** | 0.167 | 0.278 | 0.255 | 0.094* | 0.083 | 0.593 | 18.97 |
|  | **Tinned fish TM** | 0.355 | 0.018** | 0.076 | 0.626 | 0.057 | 0.712 | 5.10 |
|  | **Grilled chicken** | -- | -- | -- | -- | -- | -- | 0.10 |
|  | **Tea** | -0.04 | 0.799 | 0.4 | 0.007** | 0.156 | 0.319 | 17.93 |
|  | **Coffee** | 0.031 | 0.841 | -0.13 | 0.4 | -0.01 | 0.941 | 9.36 |
|  | **Pizza** | -0.33 | 0.029** | -0.03 | 0.871 | -0.2 | 0.188 | 2.27 |
|  | **Fried pancake** | 0.124 | 0.421 | 0.055 | 0.724 | -0.28 | 0.065* | 10.5 |
|  | **Fried chicken** | 0.271 | 0.076* | 0.077 | 0.627 | -0.15 | 0.321 | 7.23 |
|  | **Fries** | 0.103 | 0.508 | 0.076 | 0.626 | -0.24 | 0.125 | 6.73 |
|  | **Ice cream** | 0.07 | 0.65 | -0.08 | 0.633 | 0.079 | 0.612 | 6.53 |
| **Lowest** | **Soda** | -0.3 | 0.049** | -0.06 | 0.69 | -0.1 | 0.541 | 0.59 |

^a^Empty cells contain too few levels in one of the factors for valid analysis.

* Significant at p < 0.10

** Significant at p < 0.05
